# Supplementary material for: Service availability and readiness for basic emergency obstetric and newborn care: Analysis from Nepal Health Facility Survey 2021
Source: PLoS One. 2023 Aug 17;18(8):e0282410. doi: 10.1371/journal.pone.0282410 (PMC10434927; doi:10.1371/journal.pone.0282410)
Supplement: S2 Table — (DOCX) [file pone.0282410.s002.docx]

**Supplementary Table 2: Definition of independent variables**

| **Variables** | **Categories** | **Definition** |
| --- | --- | --- |
| Ecological regions | Hill/Mountain/Terai |  |
| Location of HFs | Rural/Urban | The rural-urban classification of settings was based on the municipality types in which the HFs were situated. Facilities located in rural municipality were classified as ‘rural’ whereas facilities located in municipality, sub-metropolitan and metropolitan city were classified as ‘urban’. |
| Province | Koshi/ Madhesh/ Bagmati/ Gandaki/ Lumbini/ Karnali/ Sudurpaschim | |
| Type of HFs | Federal/Provincial hospitals/ Local HFs/ Private hospitals | The hospitals under federal or provincial government were classified as federal or provincial hospitals, facilities under local governments which include local hospital, primary health care center and health posts were classified as local facilities, and the hospitals owned by private sectors were classified as private hospitals. |
| External supervision | Performed/Not performed | Facilities were considered to have external supervision if facility staff members reported receiving of any kind of monitoring or supervision from federal, provincial, or municipal authorities and interviewer observed associated documentation in the past four months. |
| Duty schedule or call list for 24-hour staff assignment | Present/Absent | The facilities were considered to have duty schedule or call list for 24-hour staff assignment if facility staff reported presence of duty schedule or call list for 24-hour staff assignment and enumerators observed duty schedule or call list for 24-hour staff coverage. |
| Review of maternal and new-born deaths | Reviewed/ Not reviewed | The facilities were considered to have reviewed maternal and new-born deaths if facility staffs reported review maternal or newborn deaths regularly and enumerators observed associated documentation. |
| Review of client opinions | Reviewed/ Not reviewed | The facilities were considered to have performed a review of client opinions if facility staffs members reported presence of a system for determining client opinions, procedures for reviewing client opinions, and enumerator observed documentation or reports of recent reviews of client opinions. |
| Quality assurance activities | Present/Not present | HFs were considered to have performed quality assurance activities if facility staffs reported carrying out routine quality assurance activities and enumerators observed documentation of recent quality assurance activities, such as quality assurance reports, supervisory checklists, mortality reviews, or audits of records. |
| Frequency of meetings in HFs | None/ Sometimes/ Monthly | HFs that reported “no” for routine management/administrative meetings were classified as “none”, while those that stated, “monthly or more often” were classified as “monthly or more often” and those that reported “irregular or every 2-6 months” were classified as “sometimes”. |
| Number of delivery beds | Count | It includes the number of available delivery beds in the facility |
| Number of Health workers | Count | It includes all health workers except administrative and other non-clinical staffs. |
