# Supplementary material for: Service availability and readiness for basic emergency obstetric and newborn care: Analysis from Nepal Health Facility Survey 2021
Source: PLoS One. 2023 Aug 17;18(8):e0282410. doi: 10.1371/journal.pone.0282410 (PMC10434927; doi:10.1371/journal.pone.0282410)
Supplement: S3 Table — (DOCX) [file pone.0282410.s003.docx]

**Supplementary Table 3: Availability of seven signal functions**

| **Characteristics** | **Parenteral antibiotics** | | **Parenteral Oxytocin** | | **Parenteral Anticonvulsants** | | **Assisted vaginal delivery** | | **Manual removal of Placenta** | | **Remove retained product** | | **Neonatal Resuscitation** | | **All services available** | |
| --- | --- | --- | --- | --- | --- | --- | --- | --- | --- | --- | --- | --- | --- | --- | --- | --- |
|  | **n** | **% (95% CI)*** | **n** | **% (95% CI)*** | **n** | **% (95% CI)*** | **n** | **% (95% CI)*** | **n** | **% (95% CI)*** | **n** | **% (95% CI)*** | **n** | **% (95% CI)*** | **n** | **% (95% CI)*** |
| **Facilities** |  |  |  |  |  |  |  |  |  |  |  |  |  |  |  |  |
| Federal /Provincial | 84 | 94.36(87.01, 97.66) | 86 | 96.65(89.98, 98.93) | 62 | 69.64(59.19, 78.40) | 61 | 68.53(58.03, 77.42) | 71 | 79.92(70.19, 87.06) | 75 | 84.32(75.09, 90.56) | 77 | 86.55(77.64, 92.27) | 40 | 45.04(34.91, 55.59) |
| Local hospital/PHCC/peripheral | 221 | 31.11(26.18, 36.50) | 491 | 88.34(83.97, 91.64) | 48 | 3.84(2.42, 6.06) | 36 | 3.93(2.25, 6.78) | 215 | 33.92(28.73, 39.52) | 159 | 22.14(17.78, 27.21) | 183 | 26.23(21.57, 31.49) | 8 | 0.31(0.16, 0.63) |
| Private Hospital | 121 | 75.21(64.00, 83.82) | 134 | 83.06(71.08, 90.73) | 63 | 42.73(32.56, 53.55) | 55 | 32.46(23.90, 42.39) | 90 | 51.78(41.23, 62.17) | 92 | 52.14(41.54, 62.54) | 72 | 45.18(35.12, 55.65) | 20 | 10.52(6.60, 16.36) |
| **Ownership** |  |  |  |  |  |  |  |  |  |  |  |  |  |  |  |  |
| Private | 121 | 75.21(64.00, 83.82) | 134 | 83.06(71.08, 90.73) | 63 | 42.73(32.56, 53.55) | 55 | 32.46(23.90, 42.39) | 90 | 51.78(41.23, 62.17) | 92 | 52.14(41.54, 62.54) | 72 | 45.18(35.12, 55.65) | 20 | 10.52(6.60, 16.36) |
| Public | 305 | 33.25(28.42, 38.45) | 577 | 88.62(84.40, 91.82) | 110 | 6.07(4.48, 8.18) | 97 | 6.12(4.27, 8.70) | 286 | 35.47(30.42, 40.87) | 234 | 24.24(19.95, 29.12) | 260 | 28.27(23.70, 33.34) | 48 | 1.83(1.35, 2.46) |
| **Province** |  |  |  |  |  |  |  |  |  |  |  |  |  |  |  |  |
| Koshi | 87 | 39.63(27.74, 52.89) | 122 | 93.3(82.18, 97.68) | 28 | 6.97(4.40, 10.85) | 28 | 9.40(5.04, 16.88) | 77 | 43.32(30.63, 56.95) | 57 | 22.63(14.19, 34.09) | 52 | 21.59(13.30, 33.07) | 15 | 3.69(2.06, 6.53) |
| Madhesh | 60 | 59.93(38.77, 77.93) | 80 | 98.9(95.29, 99.75) | 33 | 16.61(9.88, 26.57) | 24 | 23.44(10.92, 43.33) | 62 | 83.67(66.69, 92.92) | 52 | 67.21(46.70, 82.74) | 53 | 61.43(40.72, 78.69) | 11 | 5.22(2.56, 10.35) |
| Bagmati | 82 | 33.3(23.02, 45.45) | 120 | 74.16(60.99, 84.05) | 37 | 10.73(6.28, 17.75) | 32 | 9.22(4.95, 16.53) | 71 | 33.08(22.59, 45.57) | 65 | 23.55(15.18, 34.64) | 64 | 30.68(20.56, 43.08) | 12 | 2.22(1.20, 4.08) |
| Gandaki | 41 | 27.66(16.95, 41.73) | 81 | 89.82(76.38, 96.01) | 14 | 4.28(2.36, 7.66) | 14 | 4.42(2.42, 7.91) | 33 | 18.05(10.18, 29.95) | 40 | 24.74(14.84, 38.28) | 35 | 20.87(12.02, 33.74) | 4 | 1.22(0.44, 3.38) |
| Lumbini | 64 | 34.14(23.33, 46.89) | 107 | 84.66(72.23, 92.14) | 29 | 12.23(6.48, 21.89) | 22 | 4.87(2.99, 7.85) | 58 | 35.25(24.16, 48.20) | 53 | 29.72(19.58, 42.34) | 53 | 33.35(22.45, 46.38) | 13 | 2.77(1.52, 4.99) |
| Karnali | 41 | 36.56(25.74, 48.94) | 85 | 92.35(82.37, 96.90) | 14 | 8.31(3.91, 16.76) | 16 | 5.95(2.95, 11.63) | 40 | 42.12(30.58, 54.59) | 28 | 22.57(14.12, 34.08) | 31 | 21.96(13.77, 33.13) | 6 | 1.68(0.73, 3.85) |
| Sudurpaschim | 51 | 34.33(24.80, 45.30) | 116 | 93.62(85.86, 97.25) | 18 | 5.18(2.62, 9.98) | 16 | 6.04(2.85, 12.32) | 35 | 22.54(14.77, 32.83) | 31 | 14.84(8.96, 23.59) | 44 | 29.45(20.51, 40.31) | 7 | 1.51(0.70, 3.24) |
| **Location** |  |  |  |  |  |  |  |  |  |  |  |  |  |  |  |  |
| Rural | 305 | 27.07(21.38, 33.62) | 577 | 89.17(83.60, 93.01) | 110 | 2.43(1.20, 4.88) | 97 | 3.57(1.65, 7.56) | 286 | 31.71(25.50, 38.66) | 234 | 17.92(13.18, 23.91) | 260 | 20.63(15.55, 26.84) | 1 | 0.06(0.01, 0.44) |
| Urban | 121 | 49.00(41.77, 56.27) | 134 | 86.90(80.66, 91.34) | 63 | 17.47(13.65, 22.10) | 55 | 14.23(10.94, 18.30) | 90 | 43.41(36.38, 50.71) | 92 | 37.67(31.05, 44.77) | 72 | 41.50(34.59, 48.76) | 67 | 5.74(4.37, 7.51) |
| **Ecological region** |  |  |  |  |  |  |  |  |  |  |  |  |  |  |  |  |
| Hill | 193 | 28.93(23.55, 34.96) | 376 | 86.03(80.35, 90.27) | 70 | 6.14(4.27, 8.76) | 69 | 6.02(4.11, 8.72) | 182 | 32.02(26.25, 38.40) | 154 | 19.77(15.42, 24.98) | 151 | 22.4(17.59, 28.08) | 27 | 1.58(1.06, 2.35) |
| Mountain | 56 | 36.27(25.82, 48.21) | 108 | 91.44(81.61, 96.26) | 13 | 3.74(1.69, 8.05) | 15 | 5.34(2.05, 13.17) | 40 | 29.4(19.80, 41.26) | 31 | 17.08(10.07, 27.50) | 40 | 25.02(16.33, 36.33) | 4 | 0.82(0.30, 2.25) |
| Terai | 177 | 57.9(46.66, 68.38) | 227 | 91.79(83.92, 96.00) | 90 | 20.61(14.62, 28.24) | 68 | 16.31(10.70, 24.07) | 154 | 55.74(44.87, 66.10) | 141 | 52.35(41.56, 62.93) | 141 | 53.38(42.53, 63.91) | 37 | 6.39(4.36, 9.26) |
| **Overall** | 426 | 36.45 (31.83, 41.34) | 711 | 88.2 (84.25, 91.26) | 173 | 8.87 (7.02, 11.15) | 152 | 8.13 (6.20, 10.60) | 376 | 36.72 (31.96, 41.75) | 326 | 26.37 (22.28, 30.92) | 332 | 29.56 (25.23, 34.30) | 68 | 2.49 (1.93, 3.22) |
| *n: weighted frequency; %: weighted percent; CI: Confidence Interval* | | | | | | | | | | | | | | |  |  |
