# Supplementary material for: Service availability and readiness for basic emergency obstetric and newborn care: Analysis from Nepal Health Facility Survey 2021
Source: PLoS One. 2023 Aug 17;18(8):e0282410. doi: 10.1371/journal.pone.0282410 (PMC10434927; doi:10.1371/journal.pone.0282410)
Supplement: Attachment — Submitted filename: PLOS ONE-D-23-04260 Report.docx [file pone.0282410.s004.docx]

**PLOS One Review - PONE-D-23-04260**

**February 2023**

**Robert B. Clark, MD**

Overall: Excellent analysis which provides valuable guidance to policymakers and administrators regarding gaps in maternal/newborn care. Robust review with facility capacity in Nepal, emphasizing signal functions and preparedness. Appropriate recommendations regarding gaps and lack of progress in some areas.

Major Points to Consider:

1. A major point is the terminology employed throughout the manuscript, which is confusing at times. The 7 signal functions are evaluated. The preparedness of facilities are evaluated. “Availability” and “readiness” are not consistently used with these two terms and are overlapping terms. Further, the components overlap, as with uterotonic. Clear delineations between these two groups of indicators would be helpful. Consider using “performed” or “presence” or similar to describe 7 signal functions (or always state “Availability of BEmONC signal functions” as the default). Consider using “preparedness” or “readiness (exclusively) to describe the three domains.
2. With the signal functions, a major point with the results and the discussion is the lack of analysis according to delivery burden of the facility. USAID concludes “only facilities with a case load of at least 250 births in three months have a reasonable chance to encounter the complications to provide EmONC signal functions.” It is therefore unlikely that the smaller facilities would have performed all signal functions in the last three months. The percentage of facilities with > 250 births per month will all (or partial) signal functions would be more meaningful from standpoint of addressing actionable gaps in capacity.
3. Similarly, for all the facilities with less than 250 births per month, it would be expected that they would not have performed all signal functions, but knowing the ones that have been most commonly used can still be instructive.
4. The low score of the guideline and staff domain is discussed in detail. Value could be added with a list of recommendations for the most time-efficient options for addressing this specific gap. What training or courses does NHTC offer that is a refresher for AMSTL? Or AVD or resuscitation, etc.? How can this specific gap be expeditiously addressed?
5. Similarly, the authors could select 2-3 other specific major gaps and give specific guidance on solving the problem, instead of simply stating the problem exists.
6. The conclusion lacks specificity. It summarizes the study adequately, but this summary does not significantly add to what is already known from previous studies.

Minor Points to Consider:

Abstract

- Minor grammar corrections are needed throughout the manuscript.
- Readiness score scale of 100 should be mentioned.
- Conclusions are unclear and perhaps miss the most important points.

Background

- 1^st^ paragraph – inconsistencies regarding MMR decreases
- NME should be addressed more fully, such as in the first paragraph.
- Case rate fatality seems out of place as it is not addressed elsewhere.

Methods

- Six groups – unclear if the six groups are comprised of each of the types or 6 mixed groups.
- Data collection – a comment regarding the rationale for the non-use of the other data sources would be helpful.
- Service readiness – readers can also view table 4 for a list. The supplemental information could include further information regarding items, which would be helpful, i.e., what comprises Emergency Transport? Delivery package? Does resuscitation mask also include bag?
- Monitoring or supervision – does this refer to clinical or administrative supervision?
- Statistical analysis – Is there a list of the continuous variables? Were the same analyses performed for the availability of the signal functions?

Results

- The overlap of oxytocin and injectable uterotonic is confusing, since in essentially all cases these are the same. Consider dropping uterotonic from analysis.
- The context of population would be very helpful, such as % of population who lives in Hilly or Mountain regions, % of pop. in each province.
- SNCU is not mentioned earlier as a variable.
- Due to the delivery load concern, Figure 2 and the paragraph preceding it that analyzed the signal functions were not that useful. Further, the figure following the descriptive paragraph seems a bit redundant.
- The value of number of beds and health workforce is questionable, since it has no context to the size of the facilities. Similarly, the presence of SNIC/NICU would not be expected in lower level facilities, making this observation difficult to interpret.
- Figure 4 has excellent information. Unfortunately, the wise economy of combining the data into a single figure makes it difficult to clearly see the colors of the facilities wise scores.
- Since the G-A estimation plot analysis shows little difference in the scores, Figure 5 may not be that useful. A description of the analysis may suffice.

Discussion

- The decline in signal functions is important, as are possible reasons for the decline. However, this decline is overshadowed by the lack of delivery load consideration. Is the decline still evident if only facilities with 250/month are considered?
- Comparing the signal function with other countries also requires consideration of delivery load. Bangladesh is densely populated and may not have as many small delivery units as Nepal.
- The federal and covid challenges are excellent points.

Conclusion

- The message of little progress in signal function performance since 2015 is important – if there really is not progress in the facilities with large delivery loads. This deserves more attention after reanalysis.
- The same is true for the domains of preparedness.
- The conclusion might be an excellent place to restate the most severe gaps identified, with reference to concrete potential solutions described in the discussion.
