# Supplementary material for: Service availability and readiness for basic emergency obstetric and newborn care: Analysis from Nepal Health Facility Survey 2021
Source: PLoS One. 2023 Aug 17;18(8):e0282410. doi: 10.1371/journal.pone.0282410 (PMC10434927; doi:10.1371/journal.pone.0282410)
Supplement: Attachment — Submitted filename: REVIEW_PB.docx [file pone.0282410.s005.docx]

**Please find the following to be considered for revision.**

1.In Abstract, Methods section-sentence no. 3, It would be better to use inferential statistics to cover mean, standard deviation, median and interquartile range…

2.In Abstract Section, Results-1^st^ sentence, mention is Madesh and Gandaki are Facilities or Province or what is appropriate

3.The last sentence of the result section in abstract looks more like a conclusion, could the figures from your result table be added here instead

4.In introduction, 3^rd^ paragraph, it is advisable to mention a full form for SMNH and EOC.

5.Likewise for PHCC, CHU, HP etc towards the last paragraph, please do mention their respective fullforms.

6.Introduction, last para, 3 rd line, “compared to no skill’ can be omitted

7.For Methodology section, is it possible to build a STROBE flow diagram to make things more clear at a glance.
