# Supplementary material for: Service availability and readiness for basic emergency obstetric and newborn care: Analysis from Nepal Health Facility Survey 2021
Source: PLoS One. 2023 Aug 17;18(8):e0282410. doi: 10.1371/journal.pone.0282410 (PMC10434927; doi:10.1371/journal.pone.0282410)
Supplement: Attachment — Submitted filename: Response to reviewers.docx [file pone.0282410.s006.docx]

**Response to reviewers comment**

1. Please ensure that your manuscript meets PLOS ONE's style requirements, including those for file naming.

**Response:** The manuscript ensures the PLOS One style requirements.

2. Please provide additional details regarding participant consent. In the ethics statement in the Methods and online submission information, please ensure that you have specified (1) whether consent was informed and (2) what type you obtained (for instance, written or verbal, and if verbal, how it was documented and witnessed). If your study included minors, state whether you obtained consent from parents or guardians. If the need for consent was waived by the ethics committee, please include this information.

If you are reporting a retrospective study of medical records or archived samples, please ensure that you have discussed whether all data were fully anonymized before you accessed them and/or whether the IRB or ethics committee waived the requirement for informed consent. If patients provided informed written consent to have data from their medical records used in research, please include this information.

**Response:** Thank you for the feedback. We have addressed this comment in the manuscript. This study is further analysis of NHFS 2021, and no separate ethical clearance is required for further analysis in Nepalese context. In the original survey, ethical clearance was obtained from Ethical Review Board of Nepal Health Research Council. Written informed consent was obtained from all participants after explaining about risk benefits and voluntariness of participation. [line 227-232]

3. We note that Figure 4 in your submission contain [map/satellite] images which may be copyrighted. All PLOS content is published under the Creative Commons Attribution License (CC BY 4.0), which means that the manuscript, images, and Supporting Information files will be freely available online, and any third party is permitted to access, download, copy, distribute, and use these materials in any way, even commercially, with proper attribution. For these reasons, we cannot publish previously copyrighted maps or satellite images created using proprietary data, such as Google software (Google Maps, Street View, and Earth). For more information, see our copyright guidelines: <http://journals.plos.org/plosone/s/licenses-and-copyright>.

**Ans:** Thank you for the feedback. The maps presented in the manuscript are copyright free. Map was plotted in QGIS 3.22.7-Białowieża from freely available GPS dataset of the health facilities from [www.dhsprogram.com](http://www.dhsprogram.com). We used a shape file with the EPSG:4326 projection system publicly available at the official website of Survey department of Ministry of Land Management, Cooperatives and Poverty Alleviation (Government of Nepal) (<https://nationalgeoportal.gov.np/>). The software used and source of shape files are cited in the current version of map. [Line 222-226]

**Response to comments from reviewer-1, Robert B. Clark, MD**

Overall: Excellent analysis which provides valuable guidance to policymakers and administrators regarding gaps in maternal/newborn care. Robust review with facility capacity in Nepal, emphasizing signal functions and preparedness. Appropriate recommendations regarding gaps and lack of progress in some areas.

Major Points to Consider:

1. A major point is the terminology employed throughout the manuscript, which is confusing at times. The 7 signal functions are evaluated. The preparedness of facilities are evaluated. “Availability” and “readiness” are not consistently used with these two terms and are overlapping terms. Further, the components overlap, as with uterotonic. Clear delineations between these two groups of indicators would be helpful. Consider using “performed” or “presence” or similar to describe 7 signal functions (or always state “Availability of BEmONC signal functions” as the default). Consider using “preparedness” or “readiness (exclusively) to describe the three domains.

**Response:** We have corrected and have maintained uniformity in terminologies used as per the suggestion provided. We agree that there could be alternative ways the variables could be identified and used in the study. However, we have considered the ‘Service Availability and Readiness Assessment (SARA) Manual by World Health Organization to facilitate comparison of findings with previous findings from Nepal and that from other countries.

2. With the signal functions, a major point with the results and the discussion is the lack of analysis according to delivery burden of the facility. USAID concludes “only facilities with a case load of at least 250 births in three months have a reasonable chance to encounter the complications to provide EmONC signal functions.” It is therefore unlikely that the smaller facilities would have performed all signal functions in the last three months. The percentage of facilities with > 250 births per month will all (or partial) signal functions would be more meaningful from standpoint of addressing actionable gaps in capacity.

**Response:** Thank you for the feedback. We agree that the need for BEmONC signal functions depends on the number of deliveries performed per month or three months. The study involves further analysis of Nepal Health Facility Survey, that does not have variable on number of deliveries performed per month. We made calculations based on SARA tool of WHO. However, to address the feedback, we have presented disaggregated analysis by facility type for readiness score and service availability (Table number 1, 2,3 and Figure: 2). The federal /provincial hospitals (including district hospitals) are the facilities that are exclusively supposed to have BEmONC signal functions as per the policy documents in Nepal. However, to comply with the calculations performed in Health Facility Survey Report, SARA tool and other previous publications, we also have data analysis performed at aggregate level.

3. Similarly, for all the facilities with less than 250 births per month, it would be expected that they would not have performed all signal functions, but knowing the ones that have been most commonly used can still be instructive.

**Response:** Thank you for the feedback. We agree that the need for BEmONC signal functions depends on the number of deliveries performed per month or three months. The study involves further analysis of Nepal Health Facility Survey, that does not have variable on number of deliveries performed per month. We made calculations based on SARA tool of WHO. However, to address the feedback, we have presented disaggregated analysis by facility type for readiness score and service availability (Table number 1, 2,3 and Figure: 2). The federal /provincial hospitals (including district hospitals) are the facilities that are exclusively supposed to have BEmONC signal functions as per the policy documents in Nepal. However, to comply with the calculations performed in Health Facility Survey Report, SARA tool and other previous publications, we also have data analysis performed at aggregate level.

4. The low score of the guideline and staff domain is discussed in detail. Value could be added with a list of recommendations for the most time-efficient options for addressing this specific gap. What training or courses does NHTC offer that is a refresher for AMSTL? Or AVD or resuscitation, etc.? How can this specific gap be expeditiously addressed?

**Response:** We have added some contents in the discussion part of manuscript. NHTC covers issues or skills like estimation of blood loss and prevention of PPH in AMTSL training. Similarly, NHTC also conducts other training in maternal and newborn health according to need and in consultation with Family Welfare Division. There are seven provincial health training centres and 62 training sites scattered in seven provinces in Nepal**.** NHTC could further accelerate the refresher training through coordination and collaboration with the PHTCs and training sites. Further, regular training need assessment with active involvement of Provincial Health Directorate and addressing those gaps in collaboration between NHTC, PHTC and training sites.

5. Similarly, the authors could select 2-3 other specific major gaps and give specific guidance on solving the problem, instead of simply stating the problem exists.

Response: Thank you for the feedback. We have addressed feedback in throughout the discussion section.

6. The conclusion lacks specificity. It summarizes the study adequately, but this summary does not significantly add to what is already known from previous studies.

**Response:** Thank you for the feedback, We have re-written the conclusion sections (Line: 437-442)

Minor Points to Consider:

Abstract

- Minor grammar corrections are needed throughout the manuscript.

**Response:** We have checked grammar throughout the manuscript and have made corrections accordingly

- Readiness score scale of 100 should be mentioned.

**Response:** The comment has been addressed [Line:21]

- Conclusions are unclear and perhaps miss the most important points.

**Response:** We have revised the conclusion section [Line: 49-44]

Background

- 1^st^ paragraph – inconsistencies regarding MMR decreases

**Response:** We have corrected inconsistencies in the manuscript.

- NME should be addressed more fully, such as in the first paragraph.

**Response:** Thank you for the feedback. The contents on NMR has been added in the first paragraph as per suggestion

- Case rate fatality seems out of place as it is not addressed elsewhere.

**Response:** Thank you for the feedback. It has been removed [49-60]

Methods

- Six groups – unclear if the six groups are comprised of each of the types or 6 mixed groups.

**Response:** The sentence is rephrased for clarity. The facilities were classified into six categories: Hospitals, Primary Health Care Centers (PHCC), Health Posts (HP), Community Health Units (CHUs), stand-alone HIC Testing and Counselling (HTCs), and Urban Health Centers (UHCs). [Line 135-139]

- Data collection – a comment regarding the rationale for the non-use of the other data sources would be helpful.

**Response:** Thank you for the feedback. To facilitate comparison with previous publications from Nepal and publications from other countries, we have adopted ‘Service Availability and Readiness Assessment (SARA) guideline developed by World Health Organization. SARA tool is based on NHFS and we opted for analysis of NHFS in our study. Further, NHFS being the only health facility survey in Nepal, we did not have option to include any other data sources. [Line: 102-103]

- Service readiness – readers can also view table 4 for a list. The supplemental information could include further information regarding items, which would be helpful, i.e., what comprises Emergency Transport? Delivery package? Does resuscitation mask also include bag?

**Response:** The definition of the different tracer items is made available in supplementary file 1. We missed resuscitation bag while writing but Resuscitation bag and mask are included in that item while analysis which is now edited and corrected in the manuscript [Table 2]

- Monitoring or supervision – does this refer to clinical or administrative supervision?

**Response:** Monitoring and supervision refers to both clinical and administrative supervision.

- Statistical analysis – Is there a list of the continuous variables? Were the same analyses performed for the availability of the signal functions?

**Response:** Readiness score, number of beds and number of health care workers are the continuous variables.

Results

- The overlap of oxytocin and injectable uterotonic is confusing, since in essentially all cases these are the same. Consider dropping uterotonic from analysis.

**Response:** We adopted the variables names in accordance with ‘WHO Service availability and readiness assessment manual (available at: (https://www.who.int/publications/i/item/WHO-HIS-HSI-2014.5-Rev.1) and previous publication (<https://pubmed.ncbi.nlm.nih.gov/34288943/>).

- The context of population would be very helpful, such as % of population who lives in Hilly or Mountain regions, % of pop. in each province.

**Response:** This part is now added in the study setting part of the methods. [Line108-118]

- SNCU is not mentioned earlier as a variable.

**Response:** This part is removed to reduce confusion.

- Due to the delivery load concern, Figure 2 and the paragraph preceding it that analyzed the signal functions were not that useful. Further, the figure following the descriptive paragraph seems a bit redundant.

**Response**: Thank you for the feedback. Figure has been removed as it does not yield any additional information. The redundant paragraph has also been removed.

- The value of number of beds and health workforce is questionable, since it has no context to the size of the facilities. Similarly, the presence of SNIC/NICU would not be expected in lower-level facilities, making this observation difficult to interpret.

**Response:** Thank you for the feedback. NHFS does not have variable on number of deliveries. However, we have disaggregated facilities by level (local, provincial/federal and private) which we assume clarifies the issue. Provincial and Federal level facilities are expected to have SNCU /NICU level service as per government policy.[Table 1]

- Figure 4 has excellent information. Unfortunately, the wise economy of combining the data into a single figure makes it difficult to clearly see the colors of the facilities wise scores.

**Response:** We agree that the map has been complex to understand due to loading of multiple information in the same map. We have not split the map into two to make it clearer. **(Figure 3)**

- Since the G-A estimation plot analysis shows little difference in the scores, Figure 5 may not be that useful. A description of the analysis may suffice.

**Response:** The G-A estimation plot (Figure-5) has been removed

Discussion

- The decline in signal functions is important, as are possible reasons for the decline. However, this decline is overshadowed by the lack of delivery load consideration. Is the decline still evident if only facilities with 250/month are considered?

**Response:** In order prevent overshadowing due to lack of delivery load consideration, we calculated readiness score and determine BEmONC signal function availability by type of facility (federal/provincial hospital, local health facilities and private hospitals)

- Comparing the signal function with other countries also requires consideration of delivery load. Bangladesh is densely populated and may not have as many small delivery units as Nepal.

**Response:** Thank you for the feedback. We have addressed the concern

- The federal and covid challenges are excellent points.

**Response:** Thank you

Conclusion

- The message of little progress in signal function performance since 2015 is important – if there really is not progress in the facilities with large delivery loads. This deserves more attention after reanalysis. The same is true for the domains of preparedness. The conclusion might be an excellent place to restate the most severe gaps identified, with reference to concrete potential solutions described in the discussion.
- **Response:** Thank you for the feedback. We have revised the conclusion section [Line 433-438]

**Response to comments from reviewer-2**

Please find the following to be considered for revision.

1.In Abstract, Methods section-sentence no. 3, It would be better to use inferential statistics to cover mean, standard deviation, median and interquartile range

**Response:** Thank you for your suggestion, we presented mean, SD, median and interquartile range

2.In Abstract Section, Results-1^st^ sentence, mention is Madesh and Gandaki are Facilities or Province or what is appropriate

**Response:** Thank you for the feedback, we have removed the sentences and replaced with other to address other comments from other reviewers.

3.The last sentence of the result section in abstract looks more like a conclusion, could the figures from your result table be added here instead

**Response:** Thank you reviewers for your comment, we have rewrite the last paragraph of result section of abstract [line 30-37]

4.In introduction, 3^rd^ paragraph, it is advisable to mention a full form for SMNH and EOC.

**Response:** We have added full form of the SMNH and EOC in the manuscript

5.Likewise for PHCC, CHU, HP etc towards the last paragraph, please do mention their respective full forms.

**Response:** The full forms of the different acronyms are added throughout the manuscript wherever necessary.

6.Introduction, last para, 3 rd line, “compared to no skill’ can be omitted

**Response:** Thank you, we have omitted the phrase in the current version of manuscript. [Line 91-92]

7.For the Methodology section, is it possible to build a STROBE flow diagram to make things clearer at a glance.

**Response:** we have added STROBE’s flow diagram explaining sampling strategy and sample selection. [Figure 1]

Reviewer-3

Thank you for the opportunity to review this paper on Basic Emergency Obstetric and Newborn Care in Nepal using data from the National Health Facility Survey 2021. It is an important topic and reveals low levels of health facility readiness in Nepal to provide BEmONC services by using a “readiness score” across three domains (guidelines/staff, equipment/supplies, and medicines/commodities). The paper in the current form has some notable gaps, which are flagged below. With no page numbers or line numbers, I could not give specific but have provided comments by section of the paper for the authors to consider. In the future, page and line numbers should be added to assist the reviewer.

**Response:** We have added line number in this current version of manuscript

1. Abstract: the results are hard to interpret without understanding what proportion of facilities would be expected to have all signal functions (is this out of 100) and details about readiness score.

**Response:** Methods part of abstract now contains how the score is calculated. [Line 19-23]

2. Introduction- There is not consistency on focus across maternal and newborn continuum. You start by focusing on the maternal mortality SDG, with no mention of the newborn mortality target or stillbirth target (not an SDG but is Every Newborn target), and then link EMoNC to newborn mortality in the final paragraph without referencing evidence about impact of EMoNC on maternal or other birth outcomes.- You use the term EOC without spelling this out (assuming is Emergency Obstetric Care). You may want better to define these terms and what is difference between EOC, EmONC, BEmONC, CEmONC for the broader audience of PLOS ONE.

**Response:** Thank you for the feedback. We have revised the manuscript as per the feedback.

3. Methods:
- It would help the reader to explain more about the health system and facility levels under the sample section. You list the different groups of facilities, but it is not clear what services, including BMoNC or not, would be expected at each. Also in the final sentence of this section indicates that you only considered facilities that offered normal vaginal deliveries. Does this include facilitates that office BEmONC?

**Response:** We have added about health system of Nepal before sampling section. We have included all facilities that offers delivery and newborn care services. The sentences are now rephrased for clarity. [Figure 1, line 119-126]

- More details on data collection are needed in this paper to allow the reader to understand who collected the data and how it was collected (without having to go to another report or paper). For example, under variables and measurements, you indicate “The health facility was defined to have availability of functional signal function of delivery and newborn care if the service was available and provided within past 3 months preceding the survey.” However, you have not explained how it is determined – self-reporting through surveys, observation from facility visits, etc. This is the same for the other variables and measurement sections. For example, later on page 12, you have indicated “Facilitates are said to have” – said by whom? The data collection overall needs to be clearer and added to the strengths and limitations in the discussion section.

**Response:** The details of data collection and operational definition are edited to make it more clearer. [Line 185-208, 145-153]

- Please include clearly how you calculated the readiness scores. A reader should not have to go to the WHO manual to understand scoring. You describe the scoring in the results section noting that these domains each are on a scale to 33.3333 but this belongs in the methods section. You should also explain why these domains are considered of equal weight for the full score.

**Response:** The method of calculation of readiness score is mentioned in the method section of the manuscript and the detailed process of calculation is present in the supplementary file 1.

4. Results- what is the rationale for presenting the unweighted and weighted data (Table 1&2) and what do these different data tell us? Similarly unadjusted and adjusted (Table 3). This should be well explained in methods and interpreted in the narrative of the results text.

**Response:** Unweighted analysis part is removed from throughout the manuscript.

- some of the results discussed in the narrative text are not significant and could be misleading eg “facilities having system for review of maternal and newborn deaths had better readiness score than those not having such review.”

**Response:** We have revised the results sections

- the authors should make use of supplementary files to provide further details for methods and results.

**Response:** We have added supplementary files providing details of methods and results [Supplementary file 1]

5. Discussion: The study did not assess trends and yet this is a major focus of the discussion.

**Response:** We have analyzed NHFS 2015 and compared readiness score and BEmONC services availability between 2015 and 2021. [Supplementary file 3 and 4, Line 312-318, 270-275, Supplementary file 3]

Some important literature on quality improvement for MNH care broadly and specifically in Nepal is absent in the discussion and interpretation of the results thus it is difficult to see how this work contributes to the literature of what is not known already
**Response:** Thank you for the feedback. We have revised the discussion section to address the concern

6. OTHER: General grammar and typos: there are quite a few sentences that are not easy to read as well as some minor typos (eg forgot a period at the end of the sentence). You should consider a copy edit. Additionally, not all of the acronyms are spelled out for the reader eg MOHP, ICF, PHCC, HPs, CHUs, HTCs, UHCs

**Response:** Full form for all acronyms were added throughout the manuscript.

References are not complete or in line with PLOS ONE format. Reports should include a website and access date, when available.

**Response:** Thank you for the feedback. We have revised the reference sections as per PLOS One format

Ethics statement: more details are needed - even for secondary analysis you need to add what ethical clearance was received for the primary analysis.

**Response:** The ethical statement was explained in detailed in the current version of manuscript [Line number:227-232]

Data availability: please provide the exact webpage and not just the DHS general webpage to direct readers as to the source.

**Response:** The exact website for downloading data is <https://dhsprogram.com/data/available-datasets.cfm> which is now updated in the manuscript. [Line: 445-447]
