# Supplementary material for: Service availability and readiness for basic emergency obstetric and newborn care: Analysis from Nepal Health Facility Survey 2021
Source: PLoS One. 2023 Aug 17;18(8):e0282410. doi: 10.1371/journal.pone.0282410 (PMC10434927; doi:10.1371/journal.pone.0282410)
Supplement: Attachment — Submitted filename: Response to reviewers.docx [file pone.0282410.s007.docx]

**Response to reviewers**

Reviewer #1: Several minor points can use additional consideration:

**Comment:** Interpretation of the availability of signal functions is still problematic due to the mixture of low and high delivery loads at the Local HF. Further insight is limited due to the data set available. However, the primary message of low availability is clear (line 274).

**Response**: The data presented on readiness is independent of delivery load and we have tried to clarify the issue in discussion section.

**Comment:** Tracer items**:** it is unclear whether the tracer items tracked have been selected from a larger list in the SARA manual, along with the justification for which items were included/not included.

**Response:** Thank you for your feedback. The tracer items are selected based on SARA manual, previous literature and availability of data in NHFS 2021 and NHFS 2015.

**Comment:** The extremely low percentage of Local HFs (0.3%) utilizing all 7 signal functions in the last 3 months may justify even more explanation in the discussion section.

**Response:** Thank you. We have added explanation in the discussion section.

**Comment**: Grammar: further review needed for incomplete sentences (line 93), typos (line 415), increased use of articles (the, an, a, of), font change (391).

**Response:** Thank you for your feedback. We have revisited grammar and corrected wherever necessary as per your feedback.

**Comment:** The use of the term “availability” of signal functions, even if this is WHO standard, continues to be problematic. This was most obvious in lines 342-355, where the language suggested the presence of antibiotics, etc., instead of their utilization in the past 3 months (as described in lines 162-3). In other areas as well, the “availability” of signal functions implied a presence rather than utilization.

**Response:** Thank you for the feedback. NHFS takes into account the readiness and availability of the service rather than utilization. We have corrected sentences that give meaning of utilization in the manuscript.

**Comment:** Staff training in Table 2 is not defined until the discussion section and is still not clear. Does this refer to a provider receiving all the training courses in lines 390-392 in the last 2 years, or a portion, etc?

**Response**: Thank you for your comment. The definition of the tracer items, is explained in the supplementary table 1.

**Comment:** Guidelines could also use clarification. Is this a standard set of government documents present in the facility?

**Response:** Thank you for your comment. Yes, guidelines included in the survey are meant to assess the standard set of guidelines to be available in HFs. The definition of the tracer items, guidelines is kept in the supplementary table 1.

**Comment:** Partograph in Table 2 is also unclear. Is this the presence of a partograph in a patient record?

**Response:** Thank you for your comment. The facility with blank partograph were considered to have partograph. The definition of the tracer items, partograph is placed in supplementary table 1.

**Comment:** Staff readjustment due to the pandemic could also be a factor in the loss of skill personnel in maternity wards.

**Response:** Thank you, we have included this explanation in discussion section.

**Comment:** Figure 2 – Why is Local HF manual removal separate and not included in the third section?

**Response:** Thank you for your comment. The error is fixed, and figure of highest resolution is upload in the portal

**Comment:** Overall, the figures are very helpful and instructive. Great additions.

**Response**: Thank you for your motivational words.

**Response to comments from reviewer 2**

**Comment:** In Abstract, Methods section-sentence no. 3, It would be better to use inferential statistics to cover mean, standard deviation, median and interquartile range…

**Response:** Thank you. We have revised the write up. But for the clarity of readers, we have also kept the list of descriptive statistics we used.

**Comment:** In Abstract Section, Results-1st sentence, mention is Madesh and Gandaki are Facilities or Province or what is appropriate

**Response:** It is already addressed in the manuscript.

**Comment:** The last sentence of the result section in abstract looks more like a conclusion, could the figures from your result table be added here instead

**Response:** It seems like this comment was applicable for our previous version of manuscript. We have updated the manuscript as suggested

**Comment: In introduction, 3rd paragraph, it is advisable to mention a full form for SMNH and EOC.**

Response: Thank you so much for the feedback. We have completely revised the manuscript. We have included full forms in first place they appear.

**Comment:** Likewise for PHCC, CHU, HP etc towards the last paragraph, please do mention their respective fullforms.

**Response:** Thank you. Comment has been addressed.

**Comment:** Introduction, last para, 3 rd line, “compared to no skill’ can be omitted

**Response:** Thank you. We have revised the manuscript as suggested.

**Comment:** For Methodology section, is it possible to build a STROBE flow diagram to make things more clear at a glance

Response: It is already addressed in the manuscript,

**Response to comments from reviewer 3**

**Comment:** The aim and objectives of the study are not clearly laid out. It just states that you aim to the service availability and readiness using the survey (line 93-97). This should be more specific to why you aim to do this assessment and what specific objectives you hope to achieve.

**Response:** We have tried to make the objectives more specific and included the reason for doing assessment.

**Comment:** The trend analysis is an important component of the study and it features strongly in the discussion section. It should be more integrated throughout the paper. Currently, the first mention of 2015 is in the results (line 273), where is it embedded in the text and easily missed since there are many numbers presented in the two paragraphs where it is mentioned (273-275; 312-317). It is not included in the methods section or shown in any tables/figures in the paper (only the supplementary files). Given the focus of this part of the study in the discussion section, I suggest you also add is something in the introduction that justifies why you decided to look at the trends and what you would expect based on history (e.g. steady increase), add it as a specific objective of the study, explicitly mention the trend analysis in the methods and add to the related tables in the results.

**Response:** Thank you for your insightful comment. Paper from 2015 analysis have been published and our analysis of NHFS 2021 is the first paper of its kind after Nepal moved to federal structure with major changes in the way health system is organized. In discussion section, we have compared our findings with previous publication from NHFS 2015. However, to prevent duplication with previous publications, we have confined our core analysis to NHFS 2021. We have revised contents throughout the manuscript to make it more clear and have cited source wherever there is comparison with 2015 findings in discussion section.

**Comment:** Stillbirth prevention is a main outcome of EMO. Nearly the same number of stillbirths occur each year (1.9 million) as newborn deaths (2.4 million); half of stillbirths are intrapartum and preventable with access to high quality EMO. It is a major oversight to not include stillbirth throughout in your framing of EmONC as a way to reduce preventable mortality – furthering our argument that investing in EOC is a triple return on investment preventing maternal and newborn death and stillbirth. For more information, please see the UNICEF report: <https://data.unicef.org/resources/never-forgotten-stillbirth-estimates-report/>

**Response:** Thank you for the feedback. We definitely agree that stillbirth prevention is one of the outcome of EMO. Our analysis is more focused on service availability rather than outcomes like NMR and stillbirths.

**Comment:** The method used to calculate the readiness score of health facilities implies that each item has equal weight within each domain. In reality, we know that they are not equal in their efficacy to reduce mortality. This should be discussed as a limitation especially in lieu of your results.

**Response:** We agree that each items within the domains may not have equal efficacy. We believe that, determining weightage for each items included within domain based on their demonstrated efficacy and effectiveness in Nepalese context would be a separate study of high impact. We may strive in that direction in future. However, in this paper, we have taken reference from previous publications and SARA tool in computation of composite index and score.

**Comment:** The independent variables section (from line 176) is very text heavy and hard to read. I would suggest creating a table to show the variables rather than writing it out in lengthy text. A more detailed summary could be a supplementary file.

**Response:** Thank you for your comment. We have generated table showing definition of the variables as suggested.

**Comment:** In the results section, you include too much text with specific numbers that duplicate what is in the table. It is hard to read and take away key findings. I suggest you tighten the results section text throughout and use it to show key examples or ranges.

**Response:** Thank you for your insightful comments. We have reduced text in result section and include key findings in the result section

**Comment:** Table 1 includes a key but the other tables do not. It would be helpful to add to all since it is already hard to digest the volume of numbers being shared in these tables.

**Response:** Thank you for your comment, we have added key to all tables.

**Comment:** You should make it clear what time point these results are for in tables 1-4. Given you have also looked at trends, it is not clear the year you have used for the data – this should be part of each title.

**Response:** The NHFS dataset used in each tables are mentioned now as per your suggestion.

**Comment:** Grammar issues remain throughout. For example, line 94 is not a complete sentence. A full copy edit is needed.

**Response:** Thank you for your feedback. We have revisited grammar and corrected wherever necessary as per your feedback.

**Comment:** The figures are blurry and suggest high resolution for publication.

**Response:** Thank you for your comment. The figure of the highest resolution is upload in the portal but to our knowledge, figure are slightly blur when pdf was built. Based on our past experience, they will be clear when final version of paper appear in the website as original images will be used in the process.

**Comment**: The authors neglect to reference important and relevant studies from Nepal linked to EmONC and reflect how their study contributes to the literature. Some examples are listed below but this is not an exhaustive list. There is also a wealth of literature on EmONC including reviews as well as other studies (not referenced) that use SARA to assess EOC, which are not referenced and would enrich the discussion section.Without inclusion of how your study contributes to the literature, there remains the question of "so what?" especially to readers familiar with Nepal and use of large surveys (such as SARA and SPA) to assess EmONC.

References

Banstola, A., Simkhada, P., van Teijlingen, E., Bhatta, S., Lama, S., Adhikari, A., & Banstola, A. (2020). The Availability of Emergency Obstetric Care in Birthing Centres in Rural Nepal: A Cross-sectional Survey. Maternal and child health journal, 24(6), 806–816. https://doi.org/10.1007/s10995-019-02832-2

Chaulagain, D. R., Malqvist, M., Wrammert, J., Gurung, R., Brunell, O., Basnet, O., & Kc, A. (2022). Service readiness and availability of perinatal care in public hospitals - a multi-centric baseline study in Nepal. BMC pregnancy and childbirth, 22(1), 842. https://doi.org/10.1186/s12884-022-05121-z

Lewis, T. P., McConnell, M., Aryal, A., Irimu, G., Mehata, S., Mrisho, M., & Kruk, M. E. (2023). Health service quality in 2929 facilities in six low-income and middle-income countries: a positive deviance analysis. The Lancet. Global health, 11(6), e862–e870. https://doi.org/10.1016/S2214-109X(23)00163-8

Kc, A., Gurung, R., Kinney, M. V., Sunny, A. K., Moinuddin, M., Basnet, O., Paudel, P., Bhattarai, P., Subedi, K., Shrestha, M. P., Lawn, J. E., & Målqvist, M. (2020). Effect of the COVID-19 pandemic response on intrapartum care, stillbirth, and neonatal mortality outcomes in Nepal: a prospective observational study. The Lancet. Global health, 8(10), e1273–e1281. https://doi.org/10.1016/S2214-109X(20)30345-4

Sharma, G., Molla, Y. B., Budhathoki, S. S., Shibeshi, M., Tariku, A., Dhungana, A., Bajracharya, B., Mebrahtu, G. G., Adhikari, S., Jha, D., Mussema, Y., Bekele, A., & Khadka, N. (2021). Analysis of maternal and newborn training curricula and approaches to inform future trainings for routine care, basic and comprehensive emergency obstetric and newborn care in the low- and middle-income countries: Lessons from Ethiopia and Nepal. PloS one, 16(10), e0258624. <https://doi.org/10.1371/journal.pone.0258624>

**Response:** Thank you for the feedback. This analysis is an analysis from nationally representative survey and we have sought to take reference of the article that are nationally representative with methodology comparable to NHFS 2021. In doing so, some article may have been missed. However, we further reviewed the article suggested and have cited relevant articles in our manuscript.
